# Autonomous UAV Landing and Collision Avoidance System for Unknown Terrain Utilizing Depth Camera with Actively Actuated Gimbal

**DOI:** 10.3390/s25196165

**Published:** 2025-10-05

**Authors:** Piotr Łuczak, Grzegorz Granosik

**Affiliations:** Institute of Automatic Control, Lodz University of Technology, 90-537 Lodz, Poland; piotr.luczak.2@p.lodz.pl

**Keywords:** UAV autonomous landing, UAV, depth vision, machine vision

## Abstract

Autonomous landing capability is crucial for fully autonomous UAV flight. Currently, most solutions use either color imaging from a camera pointed down, lidar sensors, dedicated landing spots, beacons, or a combination of these approaches. Classical strategies can be limited by either no color data when lidar is used, limited obstacle perception when only color imaging is used, a low field of view from a single RGB-D sensor, or the requirement for the landing spot to be prepared in advance. In this paper, a new approach is proposed where an RGB-D camera mounted on a gimbal is used. The gimbal is actively actuated to counteract the limited field of view while color images and depth information are provided by the RGB-D camera. Furthermore, a combined UAV-and-gimbal-motion strategy is proposed to counteract the low maximum range of depth perception to provide static obstacle detection and avoidance, while preserving safe operating conditions for low-altitude flight, near potential obstacles. The system is developed using a PX4 flight stack, CubeOrange flight controller, and Jetson nano onboard computer. The system was flight-tested in simulation conditions and statically tested on a real vehicle. Results show the correctness of the system architecture and possibility of deployment in real conditions.

## 1. Introduction

Unmanned Aerial Vehicles continue to grow in importance. Not so long ago, they were considered to be used only by hobbyists or specialists—mainly in the military and by researchers in academia. Widespread adoption was not yet obvious, costs were high, tools and equipment were limited, and usage in the wider economy not yet clear. This arrangement further limited UAVs to be actively researched only by more affluent militaries, universities, and hobbyists.

Currently, the barrier of entry has dropped significantly. Cheap, lightweight, mass-produced, and powerful-enough electronics in the forms of microcontrollers, Single-Board Computers (SBCs), sensors like IMUs, cameras, lidar, and many others have enabled us to use more advanced control algorithms. They have limited the reliance on the mechanical construction of UAVs. Multirotor UAVs have become widespread, cheap, and easy-to-pilot platforms where the ease of use stems not from the mechanical properties of multirotors but from accurate and fast sensors and advanced control algorithms.

UAVs are still mainly used as “cameras in the sky”, as well as for photography (both professional and by individual hobbyists), reconnaissance, mapping, and searching; as delivery devices; and for cargo drop. In recent years, UAVs have also proved that they can be used as types of precision ammunition.

Basic autonomous flight based on GPS that follows waypoints and lands at given coordinates by descending with constant velocity is known as long as we assume that we move in empty space and a landing spot is always available. These assumptions limit the usability of UAVs because we often do not have prior knowledge of the environment in which we operate. In most cases, UAVs do not land during a mission and return to the starting site after its completion, so the limited knowledge is not an issue. There are, however, use cases when operation in unknown territory is necessary either to complete a task or at least to protect UAVs from crashing. One such case can be one-way cargo delivery, where we do not have enough energy to return. Another class of cases can be summarized as standby missions, where we land in a certain place and wait for further instruction to fly to a different location. In these cases, we need to have more advanced autonomous capabilities to successfully operate in those circumstances, as the assumptions of free space and landing based on proprioception will not hold.

These advanced capabilities exist but have certain limitations. They seem to not reuse sensors that are already onboard but they often add new, dedicated ones that are used only for anticollision or landing, which leads to the situation where, most of the time, they are not used. One such example is the usage of a dedicated, downward-facing camera despite the fact that the UAV might already be equipped with a gimbal with a camera of even higher quality. Lidar sensors can be used as another example where expensive sensors are used only to ensure safety. Such solutions can be too costly for simpler constructions.

To address this issue, an algorithm is proposed that takes advantage of a motorized gimbal with a camera; these are common devices on UAVs. The only difference is the type of the camera. An RGB-D camera is used, but in order to not inflate the cost, an algorithm is developed for the low-budget camera with limited depth capabilities to keep the costs limited. Intel RealSense D435i (RealSense, Cupertino, CA, USA) is used. It is also assumed that the gimbal is capable of pointing straight down. The gimbal with the camera can be used during missions as the main image sensor.

Contributions of this research:

- A new landing procedure that is compatible with a close-range imaging sensor in the form of an RGB-D camera. The algorithm is lightweight enough so that it can be executed onboard using an SBC with limited computing power like the Jetson nano.
- Usage of the gimbal motion during the landing procedure to counter the narrow field of view of the camera.
- Joining landing spot detection with an uncooperative collision avoidance strategy. Safety is enhanced due to the constant flight path monitoring. The UAV can react to the obstacle in any step of the landing.

## 2. The State of the Art in Autonomous Landing

Academic interest in autonomous UAV landing emerged in the early 2000s, although small-scale civilian studies had already begun in the 1990s. Progress was slow because onboard computers and sensors capable of meeting the required performance standards were still heavy, costly, and scarce. Their weight forced researchers to use larger airframes; at the time, multirotor drones were rare, so most early work relied on unmanned helicopters. Helicopters offered higher propulsion efficiency and longer flight times, especially with an additional payload. Helicopters can also easily use combustion engines, which further improved their flight times. They also became commercially available earlier than multirotors. However, their intricate mechanics made them costly to purchase, operate and maintain. Public demand for autonomous UAVs was limited—interest came primarily from major aviation stakeholders, the military, researchers and a handful of hobbyists. As a result, most experiments were conducted at a few large research centers, often with military funding. One example of such activity is the PALACE programme, which produced the study referenced in [1].

The state-of-the-art overview is conducted from two complementary perspectives: first, a broad literature survey; and second, an in-depth review of key individual studies. The survey, based exclusively on earlier review articles, traces the evolution of research on landing autonomy over time. The subsequent detailed review focuses on the most influential or unconventional works, enabling closer examination of noteworthy contributions without compromising overall coverage.

### 2.1. Survey Papers

The earliest survey in this overview is Kendoul’s work [2], published in 2012 but covering progress from the early 1990s onward. The author stressed that for many years, military agencies were the main sponsors of rotorcraft research, whereas sizeable civilian markets had barely begun to form. Hardware relied almost exclusively on color cameras; lidars were still considered “emerging” due to their price and weight, and few platforms carried them. Most reported studies were purely theoretical or conducted in simulation. SLAM was labelled “very young” and essentially untested in flight; the only elements regarded as fully mature were high-altitude GNSS + INS navigation and classical flight-control loops. The author also introduced taxonomies of autonomy levels that later papers routinely cite. Larger UAVs, such as fixed-wing aircraft or helicopters like the Yamaha RMAX, were commonly used. Interestingly, the use of sonar or laser altimeters was rarely reported prior to the second half of the 2000s. The first autonomous landing can be dated to 2006 and will be described later [1].

At this stage, testing was mainly focused on ensuring that systems worked and on selecting appropriate methods for further development. Far less attention was given to evaluating how these methods would perform under adverse conditions.

The next survey appeared in 2014. Landing strategies were categorized into three groups: vision-based landing, guidance-based landing, and recovery techniques for small UAVs [3]. Most experiments relied on dedicated landing sites with distinct features and RGB cameras. Lidars, depth cameras, and other ToF sensors were almost absent, and only one neural network study was found. In the same year, a companion survey was published that deliberately extended Kendoul’s 2012 work by including fixed-wing platforms [4]. The technical landscape remained largely unchanged.

In 2016, another review focused on vision systems using artificial markers and standard color cameras [5]. Landing was treated as a classical task, but only when a conspicuous helipad was used. Notably, it was not considered a solved problem—only a known one. RGB-D cameras were mentioned once, only to note that they were heavy and short-range, while lidar-based SLAM was beginning to appear more frequently.

A survey of algorithmic techniques employed up to that point shows that SfM (Structure-from-Motion) and PCA (Principal Component Analysis) dominated; RANSAC (RANdom SAmple Consensus), stereo vision, optic flow, and other classical color- or edge-based methods appeared less frequently, while neural network approaches surfaced only sporadically.

The work in [6] serves both as a review and a presentation of the authors’ own method; a more developed version of their concept appears in [7], cited later in this chapter. The review section in [6] covers lidar- and standard-camera-based techniques and is confined to outdoor scenarios. Besides those sensors, only radar is mentioned, while depth cameras—whether stereo or otherwise—are not discussed.

The 2021 overview by Shah et al. proposed a more systematic environmental taxonomy. Landing scenarios were first divided into indoor vs. outdoor and static vs. dynamic, with each subdivision further labelled as “pad known” or “pad unknown” [8]. Sensors were grouped into camera-only, lidar-only, and hybrid packages. This is the first survey to recognize non-RGB imaging enough to place it into distinct categories, although RGB and stereo vision remained dominant. A certification-driven review published the same year shifted the focus to legal constraints, human–robot coexistence, and machine learning pipelines, but still relied primarily on color imagery and did not address night operations [9].

Finally, the 2022 survey by Xin et al. reclassified landing pads as static, dynamic on land, dynamic at sea, and “complex”; each class was further divided into marker-based or markerless approaches [10]. Structured-light or ToF depth cameras were again omitted, and nocturnal campaigns remained unaddressed. A substantial portion of the paper is devoted to describing the markers used during landing, and it also reviews solutions designed for maritime operations.

Taken together, these surveys reveal several persistent patterns. After roughly 2014, the community embraced 360-degree lidars, yet GNSS, IMU, and RGB cameras continued to form the default sensor triad. The use of 3D vision increased, but passive methods such as SfM, stereo, and classical optic-flow algorithms remained dominant. RGB-D devices and active use of gimbals were almost entirely overlooked. Very few reports integrated real-time obstacle avoidance with landing logic, addressed night or low-visibility operations explicitly, or evaluated behavior when the vehicle was above the effective sensor range.

Over the years, methods employing dedicated landing markers have remained highly popular. These markers typically take the form of distinctive daylight-visible pictograms, sometimes augmented with heating elements to allow IR cameras to guide the vehicle in darkness or poor visibility. These pictograms are usually black-and-white graphics, most often realized either as coded tags (e.g., ARTag, AprilTag) or as simple geometric shapes chosen to maximize detection ease and accuracy.

### 2.2. Selected Individual Contributions

An influential early demonstration of fully autonomous landing on unknown terrain is the Yamaha RMAX study described in [1], and its performance was reported in [11]. The 94 kg petrol helicopter carried a 1.1 m baseline grey-scale stereo pair, an IMU, and a SICK laser altimeter. The vehicle first homed in on GPS coordinates provided for the nominal touchdown point, then descended along a −60° glide path in four stops at 30, 24, 18, and 12 m above ground level. At each stop, the stereo module checked slope and obstacles; below 12 m, the system switched to laser-based navigation and finally landed at 2 m using inertial data and skid load cells. The method failed at night or over texture-poor surfaces, as the authors pointed out that stereo vision is highly sensitive to surface texture. The method implicitly required GNSS to initiate the maneuver.

In [12,13], experiments with full-size helicopters and 3D lidars were conducted. The landing-site assessment was split into two stages. First, laser points were classified according to clearance, slope, and overhead space; then the skid geometry of the airframe was numerically fitted to the terrain to confirm compatibility. Another lidar-centric paper proposed a quality-weighted version of RANSAC that speeds up plane extraction by about 30%, although the algorithm was validated only on archival datasets [14].

Several researchers have argued that the landing site chosen at high altitude may become unusable as the vehicle descends and smaller obstacles are detected. A complete strategy that allows the system to discard an unsafe pad mid-descent and re-plan both path and touchdown was simulated in [15]. Despite being conducted only in a simulation environment, such complete approaches are rare. A complementary idea, namely an always-running landing spot detection, was introduced in [7]. In this approach, landing spot detection is active throughout the entire mission, and the best site seen so far is stored in memory.

Hybrid perception has also gained attention. Liu et al. fused CNN-based color-image segmentation with a 3D lidar patch-refinement stage and demonstrated the concept both in simulation and in daylight flight tests, though not at night [16].

Passive stereo SLAM methods such as ORB-SLAM2 and ROVIO have been shown to support GNSS-denied navigation and final approach at low altitude [17,18,19].

Other authors explored the use of external data. Mukadam et al. classified satellite imagery with an SVM (Support Vector Machine) to preselect potential pads [20], whereas Watterson et al. compared DSM (Digital Surface Model) and DTM (Digital Terrain Model) layers so that lidar mapping in flight only needed to fill in fine details [21]. DTM had to be taken from a database (in this case, the UK’s Environment Agency), whereas DSM could be generated online with a lidar sensor.

Compact RGB-D cameras have been tried in navigation rather than landing proper. A notable example is the fusion of 2D and depth data based on ORB features with BRAND depth descriptors. The Intel RealSense D435i was used, which remained robust under degraded visibility and demonstrated the feasibility of low-cost depth sensing [22]. Gimbal control was not used. Similarities to this work can be seen in [23], although it was simplified by using a full lidar instead of an RGB-D camera, and gimbal control was again not used.

Among purely vision-based studies of unknown terrain, several exploit optic flow. Cheng [24] used OF only for state estimation and control, and OF also underpins [25]. Texture-driven classifiers of 2D color images were introduced in [26], while the first CNN solution appeared in [27] but required large, sunlit grass patches. A Gabor-filter variant in [28] segmented smaller zones and thus relaxed that constraint. A fixed-wing project [29] reconstructed candidate sites stereoscopically from motion-induced image pairs. The hybrid CNN + OF detector of [30] ran from approximately 100 m AGL, though only offline, and resolved approximately 5 × 5 m spots. Monocular depth estimation was attempted in [31]; despite large depth errors, its output was temporally smooth enough to outline flat landing areas. In a related line of work, YOLOv7 detections from a stereo rig were inserted into an octomap; free cells after obstacle dilation indicated feasible landing spots [32].

Marker-based systems form a large sub-field in their own right. A purpose-built marker simplifies guidance because its engineered features stand out from the environment. The beacon need not be purely visual—it may use RF, infrared, thermal signatures, or multi-modal combinations. Active beacons further extend operability to poor visibility and to sensor suites beyond standard cameras. Classical pad detection with ARTag or AprilTag dominates fixed pads [33,34,35], while similar techniques enable landings on moving ground vehicles [36,37] or even alongside a standing person taken as a human “marker” [38]. One study showed how a vehicle could land on a pad whose symbol had been memorized in advance [39]. Night-time marker detection was addressed by applying image enhancement and multi-scale templates under 5 lux illumination [40] or by adaptive thresholding of black-and-white patterns [41].

Some teams reversed the paradigm entirely; an infrared stereo system or a scanning lidar on the ground tracked the airborne vehicle and radioed corrective commands, allowing precise capture without onboard vision [42,43,44].

Beyond landing itself, ancillary research tackles high-rate 3D mapping on embedded processors [45], autopilot architectures that guarantee a safe action even after major failures [46], and the certification framework required for commercial use [47].

### 2.3. Synthesis and Open Questions

Across three decades, the preferred airframe for vertical take-off and landing has migrated from planes and helicopters to electric multirotors. Lidars with hundred-meter range are now common, yet they remain heavier, bulkier, and costlier than structured-light or ToF RGB-D units, which paradoxically are still almost absent in publications on this topic. Newer lidars like the Livox 360 partially solve the high-cost problem while being relatively lightweight and having longer range, but they cannot provide RGB images and their point density is lower than that of depth cameras.

GNSS, IMU, and color cameras are firmly entrenched; additional altimeters are reported only sporadically. On the algorithmic side, classical tools such as SLAM, structure-from-motion, PCA, RANSAC, and optic flow dominate, while convolutional networks and monocular depth estimation are gaining traction. Landing strategies seldom treat the complete descent envelope as a unified problem, and hardly ever blend site selection with real-time collision avoidance or consider the effect of moving obstacles that could intrude on the pad after it has been cleared. Night trials, adverse weather, or deliberate loss of GNSS remain the exception rather than the rule.

These gaps suggest several fruitful directions for future research: exploiting lightweight RGB-D or multi-sensor gimbals to enlarge the vehicle’s field of view; integrating obstacle-avoidance logic with dynamic re-assessment of the touchdown site; devising procedures that remain safe when GNSS is unavailable or daylight is absent; designing systematic test campaigns that extend from high-altitude approach down to the final meters of flare and touchdown. In the future, motorized RGB-D systems combined with Livox-like lidars might prove to be a promising sensor combination, as lidar can significantly enhance collision avoidance while also functioning as an altimeter.

## 3. Proposed Procedure

### 3.1. Reasoning

Autonomous landing presents two intertwined challenges; the aircraft must first travel safely toward a touchdown zone that may lie beyond the depth sensor’s range, and then execute the final touchdown with sufficient accuracy.

Even when the intended landing point is outside the onboard depth camera’s range, the same camera can be used to ensure collision-free cruising—provided we know which part of the observed point cloud lies along the vehicle’s velocity vector. Most published systems either mount sensors rigidly beneath the fuselage or rely on panoramic lidars. A nadir-looking camera cannot inspect the space ahead and thus cannot protect low-altitude cruise. A 360° lidar provides omnidirectional vision, but its cost, mass, and lack of RGB imagery can limit practical use.

In most outdoor missions, the environment is quasi-static; the main threat is that the drone itself flies into a tree, mast, or wall—not that something hits the drone. Hence, the collision-checking task can be reduced to analyzing the corridor directly in front of the motion direction. If the payload includes a gimbal that can rotate the camera so its optical axis aligns with the instantaneous velocity vector, the platform gains an inexpensive “look-ahead” shield. By throttling forward speed—especially close to the ground, where danger is greatest—even a short-range depth camera provides enough reaction time. Speed control thus becomes a built-in safety layer.

A landing site may be selected from high altitude using a long-range lidar or from a few meters away using a less capable sensor. The second option inevitably increases collision risk, again highlighting the importance of an integrated anti-collision module. Even with long-range lidar, collision risks remain, as the sensor may lack the resolution to detect smaller obstacles at full range. The angular size of an obstacle may be smaller than the lidar’s angular resolution, or only a few points may register—potentially filtered out during point cloud processing.

Before any translation command is issued, the vehicle should visually sweep the intended path to ensure the flight corridor is clear; otherwise, the first meter of motion could result in an impact. In this way, the gimbal plus camera can mimic lidar by enlarging the effective field of view—a decisive advantage at close range, where each frame captures only a small slice of terrain. To use this advantage, an appropriate gimbal control strategy is required. If a multirotor is used, this strategy may also involve UAV rotations—specifically, the yaw angle can be adjusted with the UAV’s motion, though this is not true for the pitch angle.

Adding a downward-facing altimeter offers a lightweight sanity check on camera health. The only requirements are that its range exceeds that of the camera and that its “out-of-range” condition is unambiguous. If the camera link is lost or the gimbal jams, the altimeter still provides a reliable height measurement. Surfaces that fool both sensors—such as soft mud or calm water—are rare enough to be treated as corner cases; shallow puddles can be managed with a sufficiently tall undercarriage.

### 3.2. Procedure

These considerations lead to a tightly coupled architecture in which landing-site detection and collision avoidance share information.

**Figure 1:**
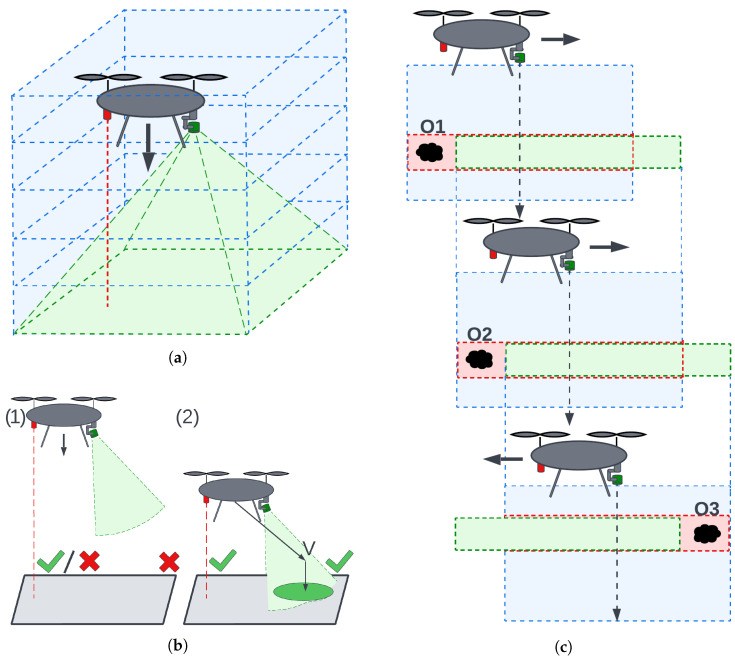
Landing procedure: (**a**) anticollision corridor, (**b**) landing phases (**1**)—descend, (**2**)—spot detection and landing, (**c**) anticollision behavior on descend. O1–3—obstacles. Blue areas—flight corridors, green cones—camera FOV, red dashed line—altimeter measurement. Green check mark on (**b**)—either altimeter or camera in measurement range. Red cross—sensor out of range. Green boxes in (**c**)—free space that avoids obstacles, Red boxes in (**c**)—collisions with obstacles.

The overall landing procedure is divided into two logical phases (Figure 1b).

During the descent phase the prospective pad lies outside the depth camera’s range (case 1 in Figure 1b). The gimbal keeps the camera pointed vertically downward, and the aircraft follows a slow, vertical path. Due to the camera’s field of view and the motion occurring along the camera’s optical axis, the anti-collision module monitors the volume along and around the flight path (Figure 1a). If an obstacle is detected, a minimal safe translation is calculated. The UAV performs a lateral translation to safely avoid the obstacle and continues descending. The magnitude of this translation corresponds to the minimal safe lateral displacement times a safety margin; the direction is chosen to minimize displacement. This procedure is illustrated in Figure 1c.

Touchdown Phase starts once the pad enters measurable range (case 2 in Figure 1b); both landing-site evaluation and collision checking run continuously. Landing-site evaluation seeks a planar surface of appropriate size and inclination, as close as possible to the point directly below the UAV. After identifying a candidate site, the camera is pointed at its center.

**Figure 2:**
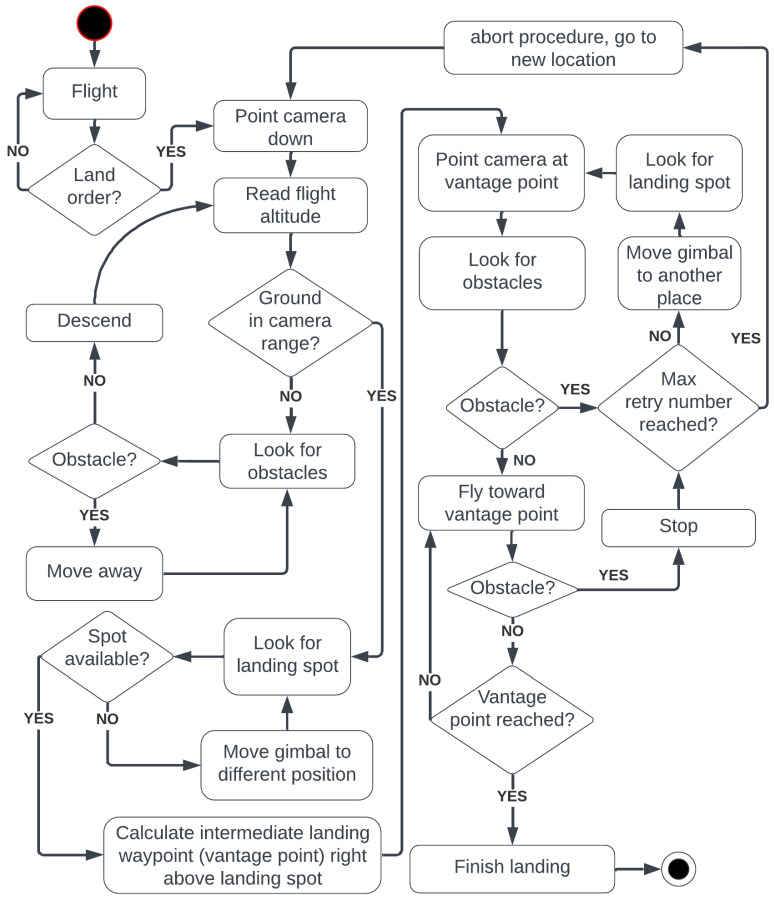
Diagram of the proposed landing procedure.

If detection remains stable across multiple frames and the flight corridor is clear, motion toward the vantage point directly above the landing site begins. If the corridor is cluttered, the anti-collision module calculates a new gimbal orientation, pans to a fresh sector, and restarts landing-site detection. If the point proves unstable, the UAV searches for a new landing site farther away. A diagram of this algorithm is presented in Figure 2.

During motion, landing-site detection is paused, but the anti-collision module continuously monitors the space along the flight path, and the gimbal orientation is continuously adjusted to point at the vantage point.

After reaching the vantage point, the final step is performed. Image processing is no longer active, the UAV is very close to the landing site, and the final touchdown occurs.

If an obstacle enters the flight corridor during the approach to the vantage point, the anti-collision module engages, motion stops, and landing-site detection restarts. The process iterates until a clear direction is found or a timeout aborts the attempt.

The two phases need not follow a fixed order. If the mission starts within camera range, the sequence begins directly with the touchdown phase; conversely, if a pad is rejected and the drone climbs above the depth horizon, control reverts to the descent logic.

In the proposed framework, the rangefinder is supervisory rather than essential. Mounted rigidly under the chassis, it always points downward and may therefore look outside the camera’s field. A known body-frame transformation converts the scalar range to an approximate footprint in the image. If camera reliability is high, the altimeter becomes optional.

## 4. Materials and Methods

### 4.1. UAV Construction

Since the proposed procedure has been implemented using a real UAV and laboratory experiments are reported in this work, its construction is also described.

The UAV used is a standard hexacopter based on the DJI F550 frame. The frame diameter is approximately 550 mm. Additional specifications can be found in [48].

**Figure 3:**
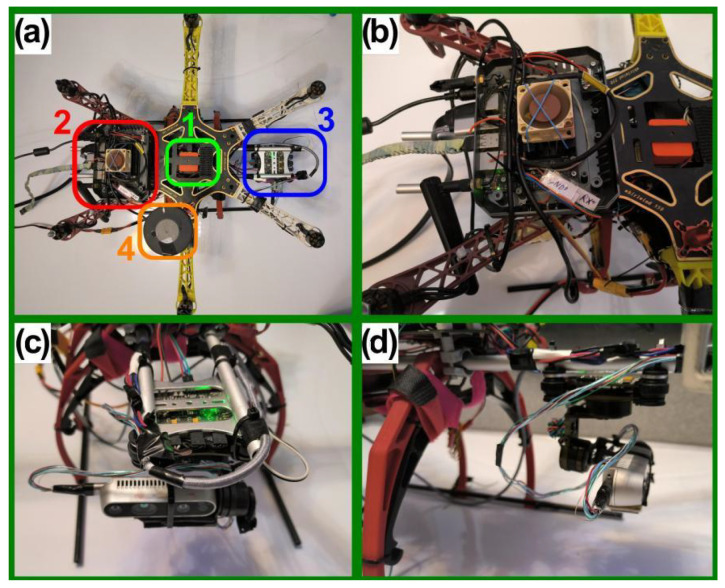
Autonomous UAV used in research: (**a**) Top view and main components. 1—Flight controller, 2—Onboard computer, 3—Gimbal with camera, 4—GPS. (**b**) Jetson nano onboard computer. (**c**) Gimbal with Intel RealSense D435i. (**d**) Side view of the gimbal mount.

**Figure 4:**
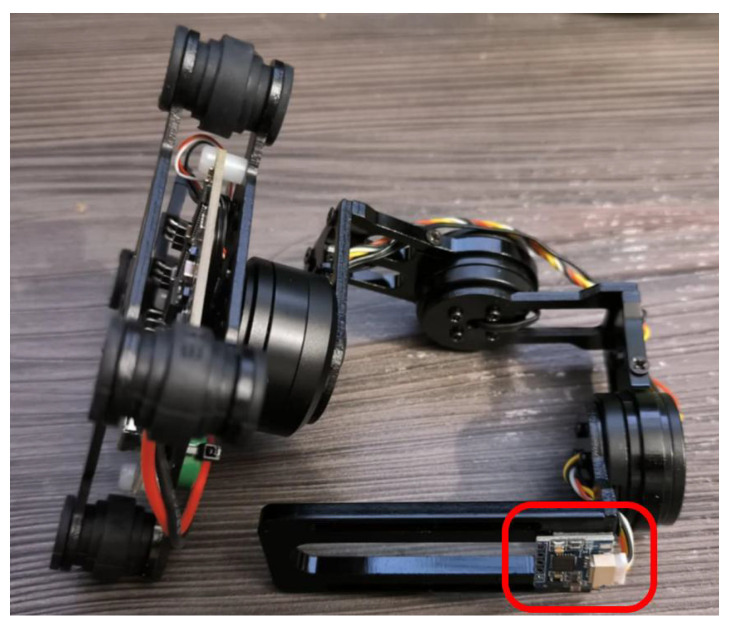
Storm32 BGC V1.32. Marked in red rectangle is IMU used by gimbal controller to calculate orientation of the work surface.

Figure 3 shows the UAV in its laboratory testing configuration. The propellers are removed, electronics are powered via a cable, and the onboard computer is not rigidly strapped to the frame to allow easier access. In Figure 3a, we can see the main components:

1. Flight controller (green rectangle): Pixhawk 2.1—CubePilot Cube Orange on a carrier board [49].
2. Onboard computer (red rectangle): Nvidia Jetson Nano 4GB A02 [50].
3. Gimbal with RGB-D camera (blue rectangle): Storm32 BGC V1.32 gimbal [51] and Intel RealSense D435i [52]. The gimbal is shown in more detail in Figure 4.
4. GPS antenna (orange rectangle). HEX/ProfiCNC Here2 GPS [53]—now discontinued, with newer versions available.

**Figure 5:**
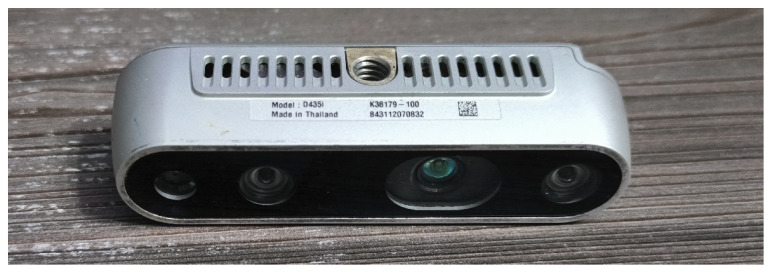
Intel RealSense D435i camera.

The Intel RealSense D435i camera (Figure 5) is a hybrid stereo RGB-D camera. Depth perception is provided by a stereo pair of Near-Infrared (NIR) OmniVision OV9282 sensors, coupled with an IR laser structured-light projector [52,54]. This gives the camera its hybrid functionality; in daylight, where the IR laser is turned off or blinded by sunlight, the camera operates as a classic passive stereo vision device. However, in low-light conditions or when the scene lacks texture, structured lighting provides the missing data, enabling operation at night.

Additionally, NIR and SWIR cameras, in contrast to visible-spectrum cameras, are less sensitive to changes in illumination, shading, fog, or dust [55].

**Figure 6:**
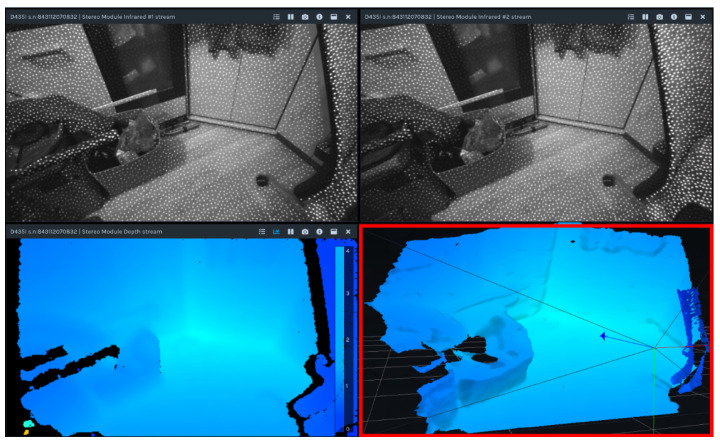
Example screens from the Intel RealSense Viewer software 2.53.1. The top images display NIR IR images from the stereo pair, with white dots indicating the effect of IR structured lighting. The bottom left image shows the depth map, and the bottom right (red rectangle) shows the resulting point cloud.

While the output of the Intel RealSense D435i is noisier and more error-prone compared to laser scanners like Ouster or Velodyne, it is more robust than passive stereo cameras. It continues to function in a broader range of environments due to its use of the NIR spectrum and structured lighting. Its compact size also allows it to be mounted on a relatively small gimbal, helping to overcome field-of-view limitations. Example image acquisition is illustrated in Figure 6.

In tasks such as autonomous landing, uninterrupted data streams may be more valuable than high precision. Since detailed mapping or advanced segmentation is not required, the focus is on classifying one class—the landing spot—and treating all others as obstacles.

**Figure 7:**
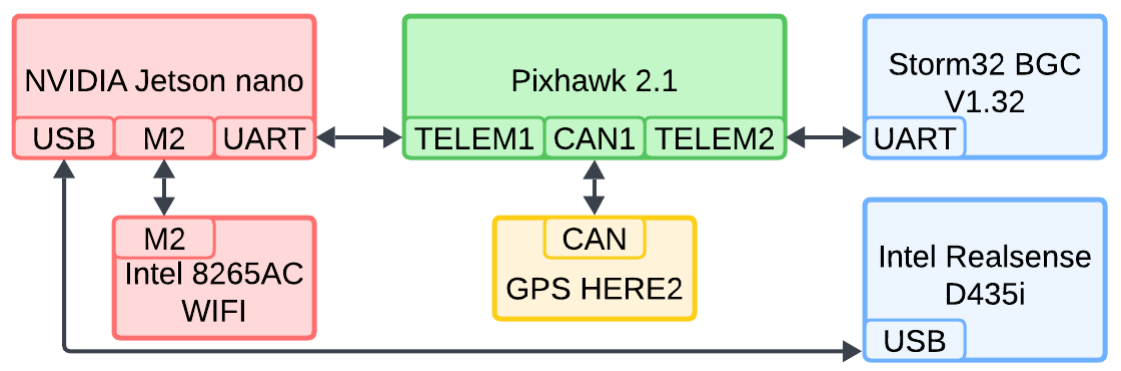
Simplified diagram of the electrical connections. TELEM1 and TELEM2 are serial port names of the Pixhawk 2.1 carrier board. Colors are kept consistent with those used in Figure 3.

The electrical connections are simple and illustrated in Figure 7. The Jetson Nano communicates with the CubePilot Orange flight controller via a serial UART connection (TELEM1 port on the carrier board). The flight controller communicates through its carrier board. The gimbal is controlled by the flight controller, which relays signals from the Jetson Nano. The GPS is connected via a CAN bus. The camera is connected directly to the flight controller via USB. The cable was modified to avoid limiting the gimbal’s range of motion. The gimbal has a yaw range of 180 degrees in both directions and a pitch range from approximately 20 degrees (looking up) to 90 degrees (looking down).

### 4.2. Software Architecture

**Figure 8:**
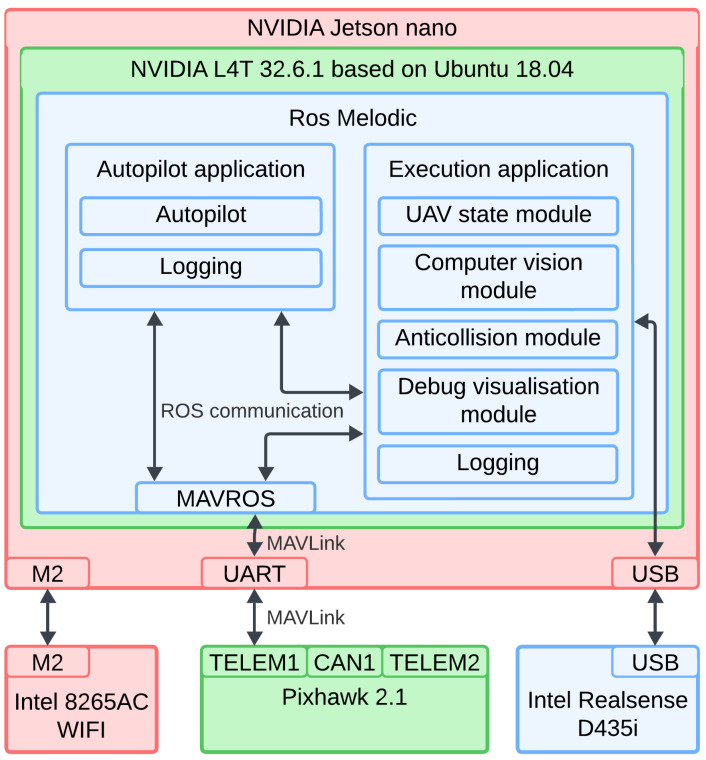
Software and hardware cooperation. Software blocks are shown in the wider context of ROS, operating system and Jetson nano SBC itself. Colors of the hardware components are kept consistent with those used in Figure 3. Colors of NVIDIA L4T 32.6.1 and Ros Melodic are added just for visual aid.

This subsection clarifies how the software communicates with the hardware. Individual software components will be explained in dedicated subsections. A diagram of the software architecture is shown in Figure 8.

The Jetson Nano used in this work runs NVIDIA L4T 32.6.1, an Ubuntu-based operating system maintained by NVIDIA, based on version 18.04 LTS. Since NVIDIA does not always use the latest Ubuntu versions for L4T releases, ROS Melodic was used. The developed software is compatible with newer versions. For simulation tests, a PC running Ubuntu 20.04 LTS and ROS Noetic was used.

The flight controller uses the PX4 flight stack, which supports installation on both flight controllers and PCs [56]. On a PC, PX4 can be used with simulation environments—Gazebo was used in this work. Communication between the Jetson Nano and the flight controller is via UART serial port using the MAVLink protocol. MAVLink messages are not automatically recognized as ROS topics. To bridge this gap, the MAVROS package was used, which translates MAVLink messages into ROS topics and handles data conversion between MAVLink and ROS conventions.

The proposed solution is divided into two separate applications:

Autopilot application: Serves an auxiliary role, providing motion execution, basic movements, and aiding in testing.

Execution application: The main software component, implementing the landing procedure and point cloud processing logic.

Both applications are written in C++. Libraries used include OpenCV, PCL, and VTK for image and point cloud processing and visualization. The execution application has a modular structure, with each module explained in its own subsection.

### 4.3. UAV State Module

This module is responsible for managing the UAV’s pose and coordinate systems. Since the gimbal is actively controlled in this work, the poses of all relevant system components must be known. To achieve this, a kinematic chain describing the UAV’s structure has been defined and is maintained within the UAV State Module. This module also receives data from the flight controller to keep the kinematic chain updated.

**Figure 9:**
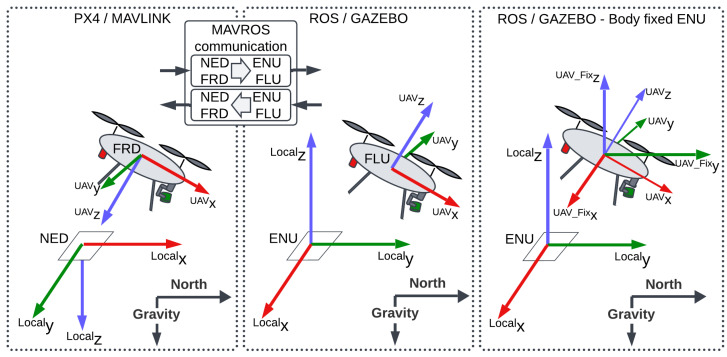
Coordinate frame conventions and conversions. MAVLink V2.0 and PX4 v1.13.3 software use the aerospace NED convention (left panel), while ROS/Gazebo use the ENU convention (middle and right panels).

The primary coordinate system used is the body-fixed ENU (East-North-Up), as landing-site location and all motions are defined as translations relative to the airframe. The body-fixed ENU frame is axis-aligned with the global ENU frame, but its origin moves with the UAV and is located at the center of the flight controller. Coordinate systems are illustrated in Figure 9.

The kinematic chain serves as the connection between the camera frame and the flight controller frame. The flight controller is solely responsible for pose estimation using an Extended Kalman Filter (EKF), while the landing algorithm handles image data processing. The kinematic chain bridges these two perspectives.

At system startup, the PX4 software on the flight controller sets the initial position as the origin of the local NED (North-East-Down) system. The EKF then updates the UAV’s position. MAVROS converts NED to ENU coordinates, which is why ENU is used instead of the aerospace-standard NED [57,58,59,60,61,62,63].

**Figure 10:**
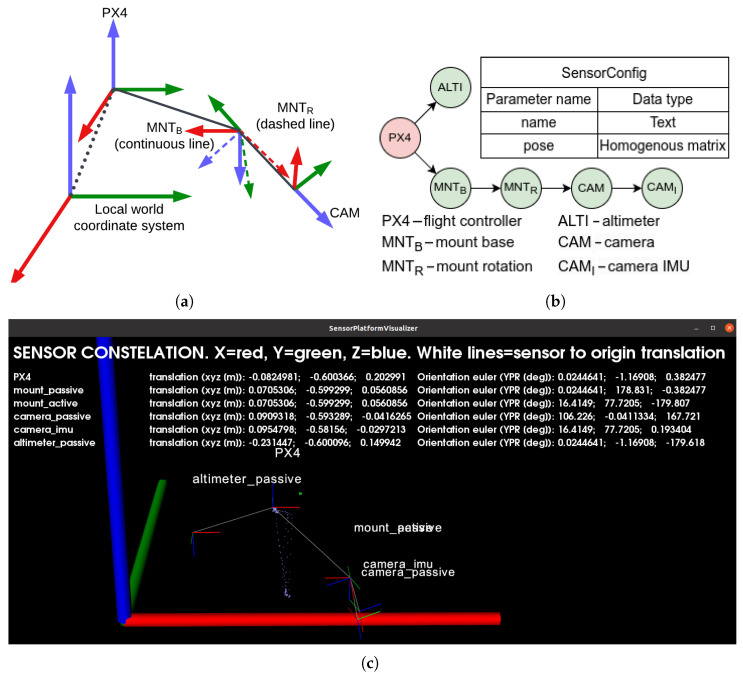
UAV kinematic chain: (**a**) Ideogram, (**b**) SensorConfig data structure—tree node, (**c**) real example together with all poses. Image generated by the debug/visualization module.

Targets are detected in the camera coordinate frame. To calculate the correct translation from the UAV to the target in the body-fixed ENU frame, we must know the transformation from the camera to the UAV frame and the orientation of the vehicle. Orientation is tracked by the flight controller and transmitted via MAVLink. The camera-to-UAV transformation is known from the kinematic chain. An example is shown in Figure 10.

Notation used: Matrices (bold capital letters) and vectors (bold lowercase letters) use left superscripts to indicate the coordinate frame in which the value is expressed, and right subscripts to indicate the transformed value. Example: ^0^**R**_1_ means the rotation of frame “1” expressed in frame “0” coordinates. Example: ^2^**p**_A_ means the position p of the point A expressed in the frame “2”.

Homogenous transformations are used. Let:

(1) $$A=\left[\begin{array}{cc} R & p\\ 0 & 1 \end{array}\right],\;\left[\begin{array}{c} r_{A}\\ 1 \end{array}\right]={\left[\begin{array}{cccc} r_{\mathrm{xA}} & r_{\mathrm{yA}} & r_{\mathrm{zA}} & 1 \end{array}\right]}^{T}$$

Given that, any point A expressed in the camera (CAM) frame can be written as rAPX4

(2) rAPX41=AMNTBPX4AMNTRMNTBACAMMNTRrACAM1

Pose of the UAV in the local world frame can be written as:

(3) APX4Local=AUAV_FixLocalAPX4UAV_Fix

Given axis-alignment of the ENU and body-fixed ENU frames, we get:

(4) AUAV_FixLocal=13x3pUAV_FixLocal01,  APX4UAV_Fix=RPX4UAV_Fix001

By combining (2) with (4), we can freely convert from the camera frame to any other frame by chaining as many transformations as needed.

Accuracy Considerations. Inaccuracies in the kinematic chain can lead to position errors or drift. However, their impact is limited due to the following reasons:

- Limited camera range: Small angular errors do not result in significant distance errors.
- No SLAM or odometry: All calculations are performed in a single frame, limiting error accumulation. The UAV does not require the precision of a robotic arm used in manufacturing.
- Flight controller stability: The flight controller, equipped with redundant IMUs, compass, and barometer, performs pose estimation using EKF and provides a stable base pose. The rest of the UAV is treated as a rigid chain. Yaw drift was minimal and did not cause issues. If yaw drift occurs, it can be minimized through manufacturer-recommended calibration procedures, including thermal calibration of IMUs.
- Landing candidate tracking: The UAV points the camera at the landing spot candidate to center it in the image. Inaccurate chain transformations may cause misalignment, but as long as the rotation direction is correct, the drift settles with an offset. Small offsets are acceptable since enough space around the landing spot remains visible.
- Gimbal movement threshold: The gimbal only moves when the landing spot is sufficiently far from the image center. This behavior limits unnecessary camera motion and reduces the impact of minor inaccuracies.

No issues were observed during simulation, as correct values could be read from the model configuration.

### 4.4. Computer Vision Module

**Figure 11:**
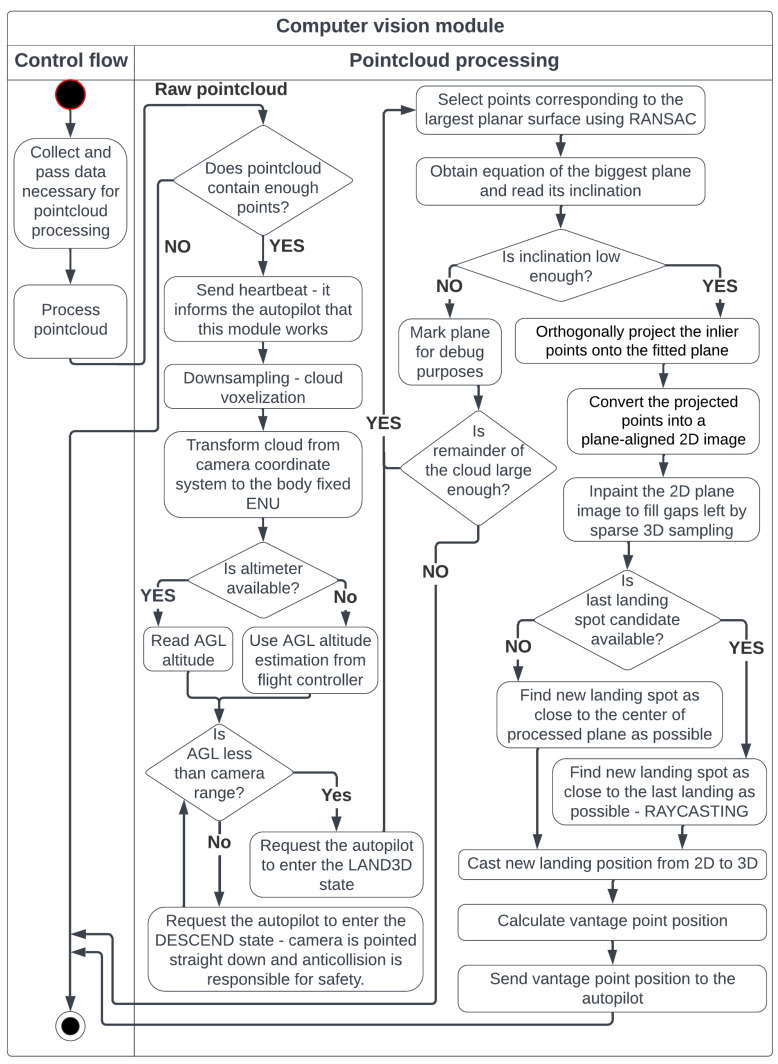
Steps of the pointcloud processing, which is the central part of the proposed solution.

This module contains the landing-site detection algorithm. The workflow is presented in Figure 11.

Landing-site detection is based on plane extraction using the RANSAC method, chosen for its speed, reliability, and availability of high-quality implementations. Implementation quality is crucial because the algorithm is intended for use during real flight. An inefficient implementation could slow down point cloud processing, even if the method itself is theoretically fast and accurate. For this reason, the RANSAC implementation from the PCL library was used.

**Figure 12:**
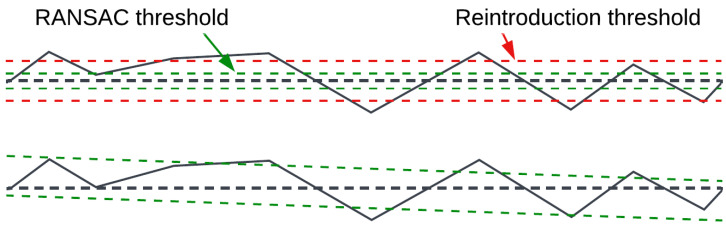
RANSAC with point reintroduction. Black dashed line—ground truth planar surface.

The RANSAC method was modified by adding an additional post-processing step—point reintroduction (Figure 12). Better results were observed when the RANSAC distance threshold was lowered and resulting holes were filled by reintroducing nearby points. Keeping the RANSAC threshold equal to the reintroduction threshold led to shifted results. This may be because the analyzed surfaces often have objects placed on them rather than holes, which shifts the results. If objects are placed evenly, the plane inclination may be preserved, but translation could occur. If objects are placed unevenly, inclination may also change, as RANSAC tries to fit as many points into the plane as possible. Since the correct plane equation is more important at this stage than having all inliers, a stricter RANSAC threshold was used, which tended to better extract the plane itself. Reintroduced points are not used to refine the plane equation; they help fill holes created by the narrow RANSAC threshold while preserving the correct plane.

When working with real surfaces (both indoor and outdoor grass), it was observed that increasing the threshold resulted in more plane candidates but reduced predictability. If the threshold is too high, false planes may be detected—for example, parts of walls or tops of obstacles may be classified as a plane despite having different heights, simply because they fit within the wide threshold. Conversely, a threshold that is too narrow results in fewer inliers, as camera noise pushes points outside the threshold value.

The best results—both indoors and on grass—were achieved with a threshold of approximately 2–4 cm. Extracted planes had holes, but the plane equations were correct. To fill the holes, the previously mentioned point reintroduction was added, using a threshold of 4–8 cm. Reintroduced points helped fill spurious holes. Thresholds must not exceed the UAV’s suspension limit, or the risk of toppling increases.

After retrieving sufficiently co-planar points, they are projected onto the plane’s equation and transformed into a 2D image, where a landing spot of appropriate dimensions is searched for. At this stage, all point cloud operations are performed in the UAV_Fix frame. Given point p from the input cloud and planar equation:

(5) p=pUAV_Fix=pxpypz,ax+by+cz+d=0

The point-to-plane distance is calculated as:

(6) $$dist=\frac{|ap_{x}+bp_{y}+cp_{z}+d|}{\sqrt{a^{2}+b^{2}+c^{2}}}$$

We also normalize the plane normal (in the following steps, we will omit the UAV_Fix superscript for brevity):

(7) n=nUAV_Fix←nUAV_Fix∥nUAV_Fix∥,where:nUAV_Fix=abc

Then, the points are projected:

(8) $$p_{x}^{\prime }=p_{x}-dist\cdot a,\;p_{y}^{\prime }=p_{y}-dist\cdot b,\;p_{z}^{\prime }=p_{z}-dist\cdot c$$

Before creating the 2D image and detecting the landing spot, we must be able to transform between 2D and 3D coordinates. To do this, we assign a frame to the detected plane, called Land_plane, defined as:

(9) ALand_planeUAV_Fix=RLand_planeUAV_FixpLand_planeUAV_Fix01

where:

(10) pLand_planeUAV_Fix=p¯=p¯xp¯yp¯z

with:

(11) $${\bar{p}}_{x}=\frac{1}{\left|L\right|}\sum_{p\in L}x,\;{\bar{p}}_{y}=\frac{1}{\left|L\right|}\sum_{p\in L}y,\;{\bar{p}}_{z}=\frac{1}{\left|L\right|}\sum_{p\in L}z$$

Let vector u define the UAV’s up direction. The axis and angle of rotation are:

(12) w=u×v,cosθ=u·v

Define the rotation quaternion:

(13) $$q_{w}=\cos\left(\frac{\theta }{2}\right),\;q_{v}=w\cdot \sin\left(\frac{\theta }{2}\right),\;w=\frac{w}{∥w∥}$$

(14) $$q=\left(q_{w},\;q_{x},\;q_{y},\;q_{z}\right)=\left(\cos\left(\frac{\theta }{2}\right),\;w_{x}\sin\left(\frac{\theta }{2}\right),\;w_{y}\sin\left(\frac{\theta }{2}\right),\;w_{z}sin\left(\frac{\theta }{2}\right)\right)$$

Then:

(15) RLand_planeUAV_Fix=1−2(qy2+qz2)2(qxqy−qwqz)2(qxqz+qwqy)2(qxqy+qwqz)1−2(qx2+qz2)2(qyqz−qwqx)2(qxqz−qwqy)2(qyqz+qwqx)1−2(qx2+qy2)

Substituting the translation (10) and (15) rotation equations into the transformation matrix (9) yields the required transformation. Casting and resolution adjustment is performed as follows:

(16) $$\begin{array}{cc} {\mathrm{pix}}_{x} & ={\mathrm{vox}}_{x}\cdot \mathrm{scale}+\mathrm{halfSize}3D\cdot \mathrm{scale},\\ {\mathrm{pix}}_{y} & ={\mathrm{vox}}_{y}\cdot \mathrm{scale}+\mathrm{halfSize}3D\cdot \mathrm{scale}. \end{array}$$

where:

scale—cloud-to-plane scaling factor;

halfSize3D—half of the maximum anticipated cloud width;

pix—2D pixel;

vox—3D point cloud point, expressed in the Land_plane frame.

**Figure 13:**
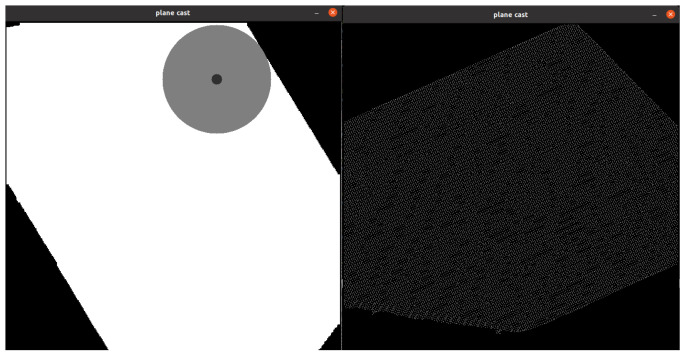
Point cloud is sparse due to the voxelization process. (**Left**)—continuous plane achieved with morphological dilation. (**Right**)—no dilation used.

**Figure 14:**
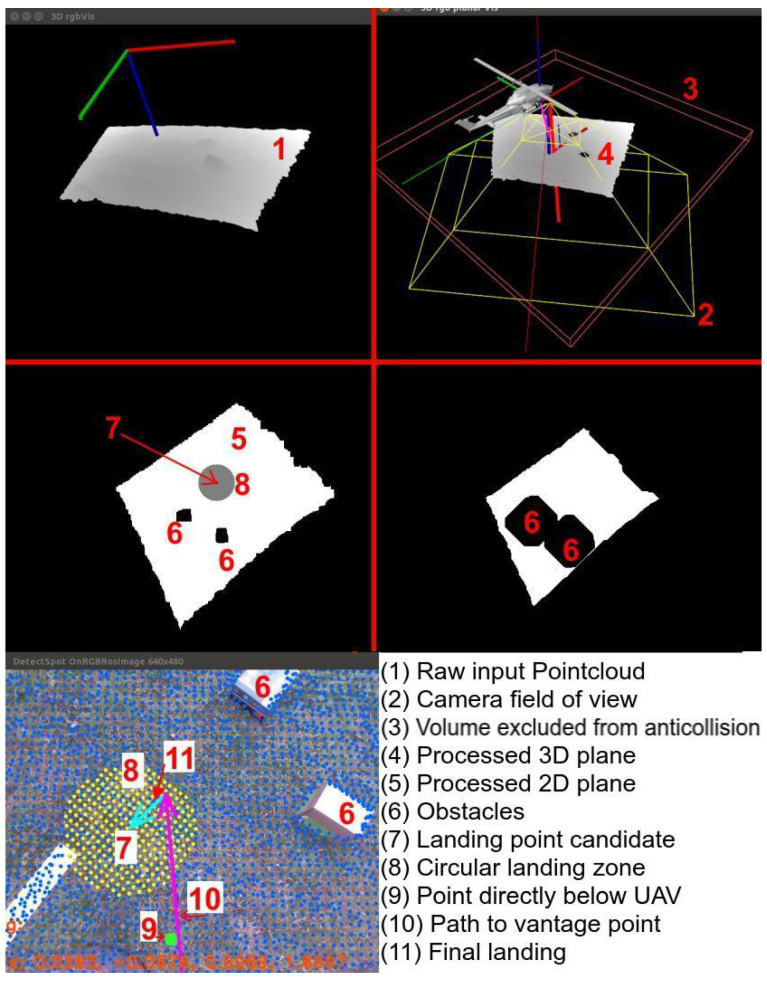
Visualization of point cloud processing. Camera depth and RGB fields of view are known, enabling augmented RGB images (bottom left). All images are from the debug/visualization module.

Due to the sparse nature of the voxelized pointcloud, morphological dilation is performed to make the plane continuous (Figure 13). Visualization of point cloud processing is illustrated in Figure 14.

### 4.5. Anticollision Module

**Figure 15:**
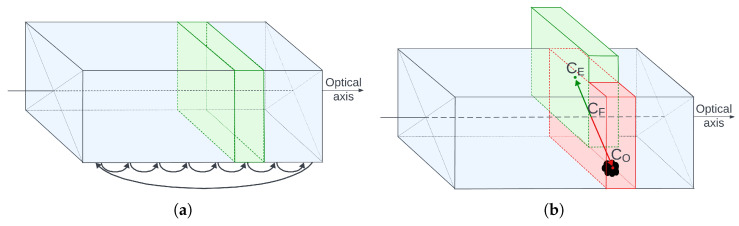
Anticollision corridor—working principle: (**a**) slice-by-slice scan pattern, (**b**) safe dislocation calculation.

This module is responsible for safety. It works by scanning the volume along the camera’s optical axis, slice by slice. If a given slice contains more than a few points, an obstacle is detected. The shape of the obstacle is simplified to its center of mass. The slices are thin enough to minimize the error introduced by this assumption. The slicing pattern is shown in Figure 15a.

**Figure 16:**
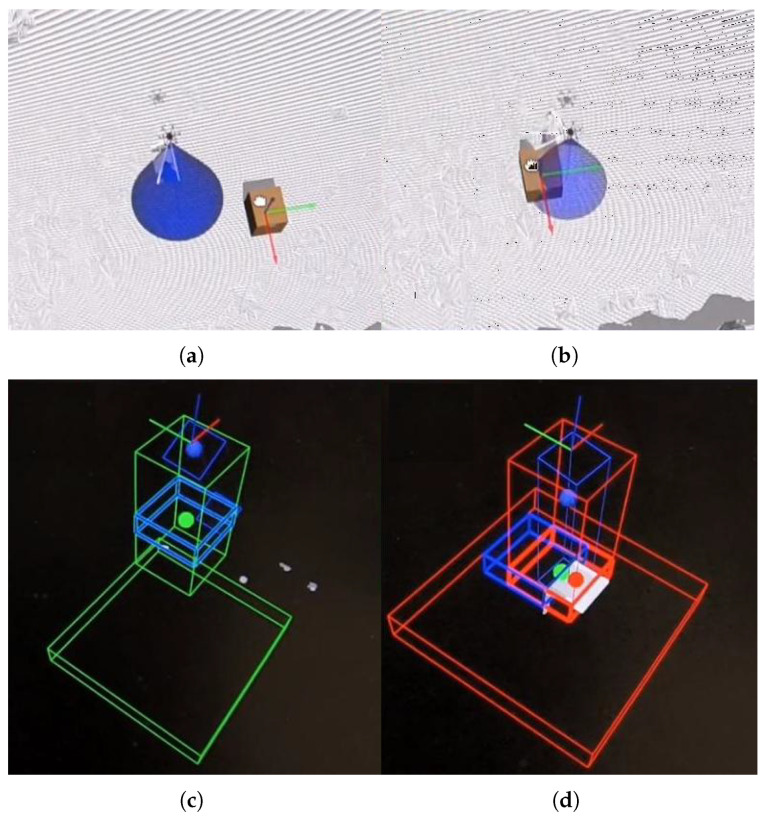
Anticollision presentation—Gazebo simulation: (**a**) no obstacle (Gazebo: box is away from the UAV), (**b**) obstacle present (Gazebo: box is in the camera’s FOV), (**c**) no obstacle (anticollision module: blue box fully inside the free (green) corridor), (**d**) obstacle present (anticollision module: blue box shifted outside the cluttered (red) corridor to avoid the box). Short and wide wire boxes at the bottom of (**c**,**d**) marks the landing surface position inside anticollision module—visual aid.

Figure 15b illustrates what happens when an obstacle is detected. Point $C_{O}$ is the center of mass of the obstacle, $C_{F}$ is the geometric center of the slice containing the obstacle (always located along the optical axis), and $C_{E}$ is the center of an empty, safe, dislocated slice. The line segment $C_{F}C_{E}$ represents the safe translation. These translations are also shown in Figure 1c and Figure 16.

The gimbal motion strategy, as described in the Proposed Procedure section (Section 3), treats the flight path as aligned with the camera’s optical axis. Therefore, the current UAV and gimbal positions define the currently checked corridor. Essentially, the camera’s optical axis serves as the basis for both motion planning and collision avoidance, paired with the rule that the camera should point at the target position before any motion begins.

To navigate in environments with obstacles, the following rule is applied. Before any motion can begin, the gimbal must point the camera in the direction of the planned motion. Then, the anticollision algorithm checks for obstacles. If an obstacle is detected during this checking phase, the gimbal rotates to move the obstacle outside the flight corridor. A new corridor is then checked, and another gimbal motion is initiated if obstacles are still present. This typically occurs during the landing spot detection phase. Once a free corridor is found, the anticollision algorithm generates a signal to restart the landing spot detection from the point currently aligned with the camera’s optical axis.

**Figure 17:**
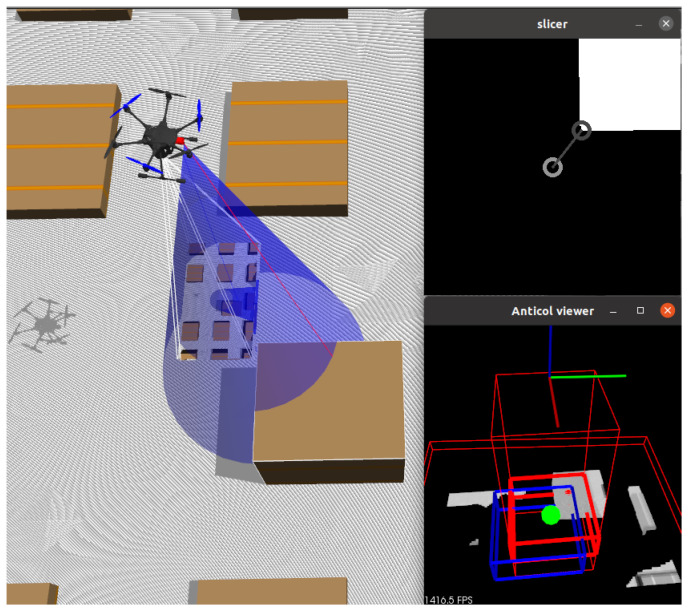
Visualization of slice point projection onto 2D. (**Left**): simulation view. (**Top-right**): slice projection. The light gray circle marks the image center; the dark gray circle marks the closest obstacle point (new $C_{O}$). (**Bottom-right**): 3D corridor visualization.

It is also possible to use the same image processing principles as in landing spot detection to account for obstacle shape. Cloud points from the corridor slice containing the obstacle can be projected onto a 2D image. This projection image has the same width and height as the collision avoidance corridor. The optical axis intersects the image at its center. Therefore, $C_{O}$ can be redefined as the point closest to the image center, and $C_{E}$ can be calculated as before. Since the number of cloud points in a single slice is limited compared to the full 3D scene, the closest point is found by iterating through all slice points. Example operation on a single slice is illustrated in Figure 17.

### 4.6. Debug Visualization Module

This module generates a visual preview of the data produced by the hardware module (SensorPlatformVisualizer) and the vision module (3Dvis). It can be enabled or disabled as needed and runs in a separate thread to minimize its impact on processing times. Offloading it to a dedicated thread is necessary because refreshing the 3D scene requires deliberate delays, during which the CPU mostly waits (VTK library, version 9.0.0 or higher; exact versions may differ between the PC and the onboard computer).

Many of the illustrations used in this work are taken from this module.

### 4.7. Autopilot

**Figure 18:**
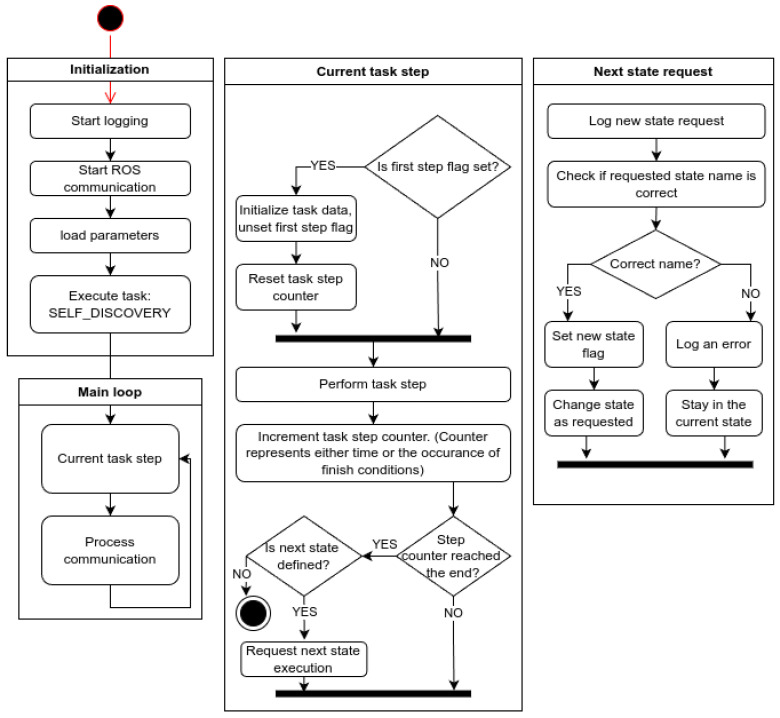
Autopilot diagram.

In this work, the autopilot is defined as the application responsible for executing motion commands. Additionally, it mimics the behavior of the UAV during a mission. While the autopilot is not the main focus of this work, its architecture is briefly introduced for completeness. Its architecture is illustrated in Figure 18.

The autopilot is designed as a finite state machine capable of simulating simple missions. The exact form of the state machine graph for a given experimental scenario is defined manually from a predefined set of behaviors.

Available states:

- **INVALID**—Pseudo-state indicating a request for an unknown state. Triggers an error log entry and keeps the current state unchanged.
- **SELF_DISCOVERY**—The vehicle listens for flight controller traffic and updates its own state; sets the home position.
- **IDLE**—Passive waiting.
- **TAKEOFF**—Takes control of the vehicle, arms the motors, and climbs vertically.
- **HOVER**—Holds position at the current location.
- **PATROL**—Flies a circle of a specified radius around the current position.
- **GIMBAL_SWEEP**—Rotates the gimbal through a full sweep.
- **GO_COORDS**—Flies to specified coordinates.
- **GO_RANDOM_COORDS**—Flies to random (nearby) coordinates.
- **GO_HOME**—Returns to the initial (home) position.
- **TRANSLATE**—Moves by an offset relative to the current position.
- **DESCEND**—Performs a slow descent; used when landing outside the camera’s working range.
- **LAND**—Commands landing according to the flight controller’s internal procedure.
- **LAND3D**—Handles vision-guided landing; listens for messages from the execution app.
- **GIMBAL_ON_LAND_SWEEP**—Service/test function; performs the gimbal-sweep procedure used during live tests.
- **WAIT_ON_LAND**—Service/test function; ground-test equivalent of **HOVER**.

## 5. Testing Environment

### 5.1. Simulation Environment

Experiments were conducted both in the Gazebo simulation and in a laboratory setting using the constructed UAV.

**Figure 19:**
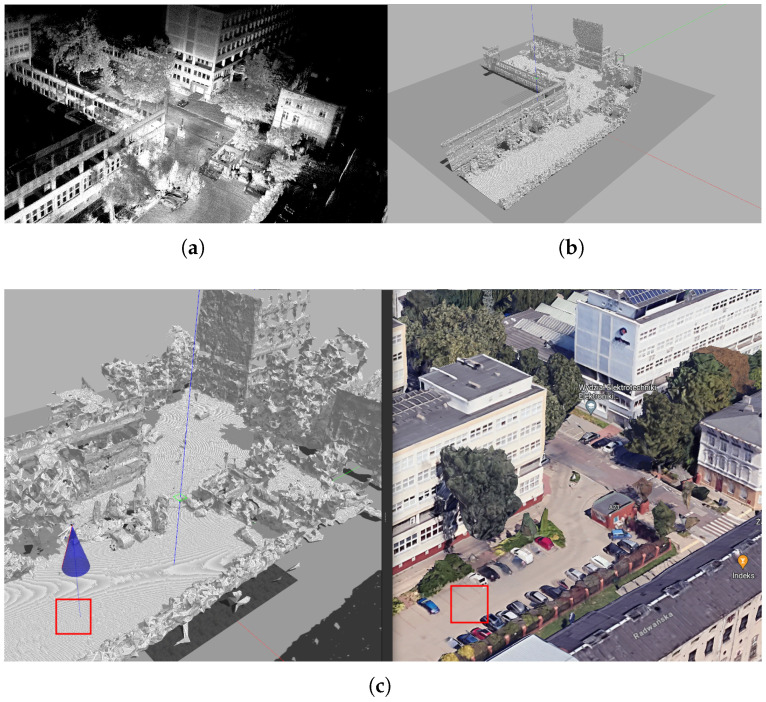
Simulation environment (Lodz University of Technology): (**a**) Registered ouster lidar scans. (**b**) Cleared and meshed scene Gazebo. (**c**) Comparison with Google Earth. The red square shows the location of the static obstacle landing tests.

To ensure that simulation testing corresponded with real-world conditions, a 3D map of the Lodz University of Technology campus was created by recording and registering 3D scans obtained with an Ouster lidar (Figure 19). While the results were not as visually refined as those in Google Maps, they preserved small local features—such as potholes, trees, individual cars, bushes, and curbs—that are typically smoothed out in Google Maps.

### 5.2. Laboratory Environment

**Figure 20:**
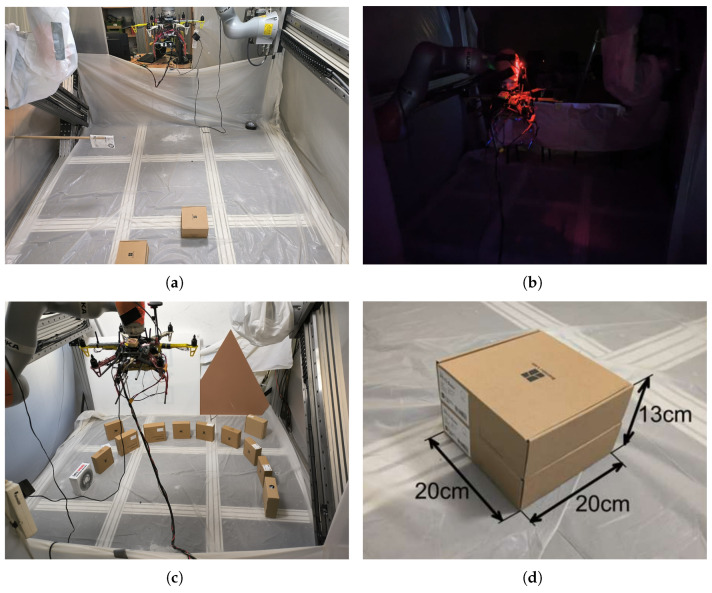
Images of the laboratory test on the real UAV: (**a**) Scenario 3, (**b**) Scenario 1—night, (**c**) Scenario 4, (**d**) Scenarios 2 and 3—obstacle dimensions.

Tests on the real UAV were conducted using a robotic arm to hold the UAV, while all sensing and processing were performed onboard (Figure 20). Batteries were removed, and power was supplied via cable. The robotic testing site is located at the Institute of Automatic Control, Lodz University of Technology [64].

## 6. Simulation Experiments

### 6.1. Landing Accuracy Check

This preliminary test ensured that the UAV could perform a sufficiently accurate landing maneuver in open space. The landing site was an empty parking lot, marked by the red square in Figure 19c.

### 6.2. Repeated Landing with Static Obstacles

**Figure 21:**
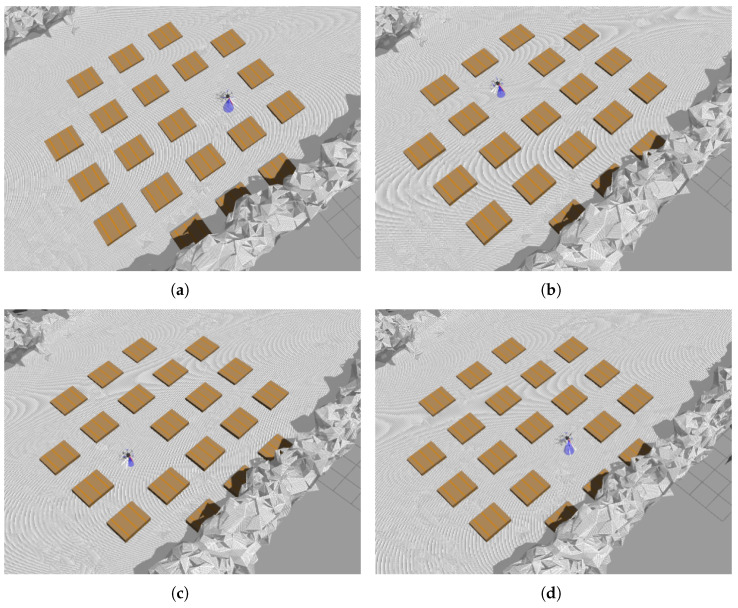
Landing in statically cluttered environment: (**a**) scenario “east”, (**b**) “north”, (**c**) “west”, (**d**) “south”. Direction “north” indicates UAV direction forward and “east” is on the right.

This experiment tested whether the UAV could find a landing spot regardless of its position relative to the airframe. Four cases were tested: “east,” “north,” “west,” and “south” (Figure 21). Collision avoidance was not triggered in this test. Obstacles were positioned so that only plane extraction and processing were active. Directions are relative to the UAV body: “north” indicates the forward direction, as estimated by the flight controller, and “east” is to the right.

### 6.3. Intrusion Test

This was the most challenging test. If previous tests failed, this one was also likely to fail. The test was conducted in a more uneven simulated terrain (curbs, nearby cars). The UAV began its descent from outside the camera’s range. Upon entering the camera’s range, a landing spot was detected directly below the UAV, as in the landing accuracy test, and the flight to the vantage point began. During this phase, an obstacle was introduced into the camera’s field of view. The UAV was expected to detect the obstacle and select a new landing spot, accounting for terrain unevenness.

**Figure 22:**
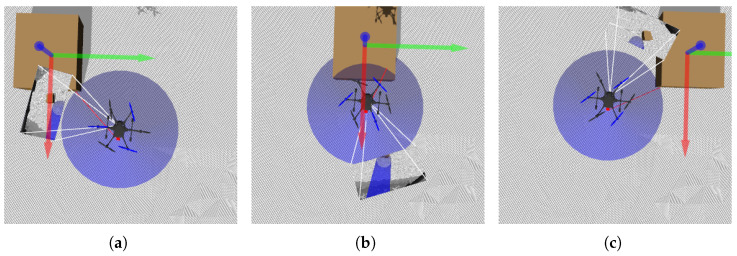

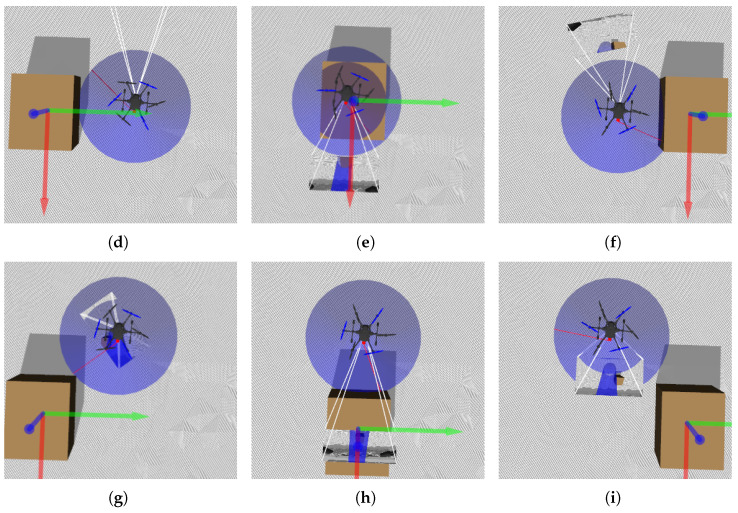
Anticollision module test—intrusions: (**a**) top-left, (**b**) top-center, (**c**) top-right, (**d**) middle-left, (**e**) middle-center, (**f**) middle-right, (**g**) bottom-left, (**h**) bottom-center, (**i**) bottom-right.

To check for potential blind spots, obstacles were introduced from all nine directions (Figure 22). The UAV’s reaction should be consistent in all cases.

## 7. Laboratory Environment

**Figure 23:**
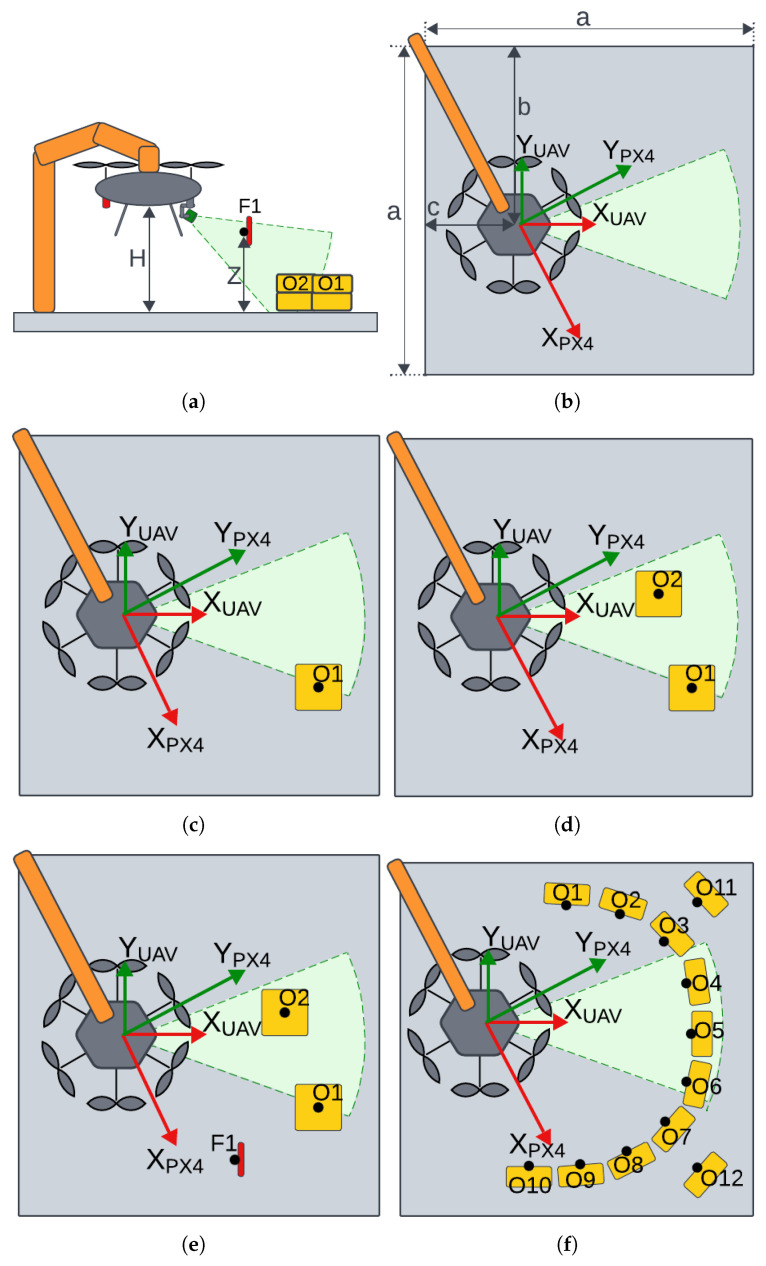
Diagrams of the laboratory test on the real UAV: (**a**) side view, (**b**) scenario day and night—no obstacles, (**c**) scenario 1—one obstacle, (**d**) scenario 2—two obstacles, (**e**) scenario 3—two obstacles with anticollision (flag F), (**f**) scenario 4—forced landing.

Similar to the simulation tests, laboratory experiments were conducted in a gradual manner, with each test building on the results of the previous one. The landing procedure was slightly modified due to height limitations—the gimbal’s starting position was not straight down (pitch = 90°) but angled (approximately 50–60°). Figure 23a,b and Table 1 show that the UAV was mounted low and toward the back of the test stand due to robotic arm and cable length constraints.

Setting the gimbal at an angle allowed a larger portion of the test stand floor to be visible, simplifying scenario setup—initial conditions could be configured in front of the UAV. It is worth noting that the gimbal angle was not fixed. Motion during landing spot detection was not restricted. Moreover, before each landing spot detection, the autopilot application took control of the gimbal, reset it to the initial position, and initiated a slow pitch sweep. The pitch sweep ranged from 20° to 70°, always starting at 20°. Due to system delays introduced to allow the gimbal to reset and begin sweeping, the effective gimbal angle at the start of landing spot detection was around 50–60°.

- **Scenario Day/Night—Change in Lighting**
  Preliminary Test. This test checks whether the system can operate effectively in both day and night conditions. The system is considered illumination-invariant if the landing sites identified under both lighting conditions overlap. This test also serves as a repeatability check—demonstrating the system’s tendency to prefer similar landing sites in similar environments, resulting in clustering. The persistence of this effect was verified on the real UAV (Figure 23b).
- **Scenario 1—Single Obstacle**
  The approach point identified during the day/night/repeatability tests is now obstructed by a single obstacle. The vision algorithm must reject this point and select a new landing site (Figure 23c).
- **Scenario 2—Two Obstacles**
  The new landing site chosen in Scenario 1 is now blocked by a second obstacle. The algorithm must relocate the landing site to a third, distinct position (Figure 23d).
- **Scenario 3—Two Obstacles with Collision-Avoidance Trigger**
  Both previous obstacles remain, and a hanging marker is placed near (but not covering) the Scenario 2 landing site to trigger the anti-collision module. The system must select yet another, entirely new landing site (Figure 23e).
- **Scenario 4—Forced Landing Zone**
  Obstacles are arranged from the beginning to constrain the free area, forcing the algorithm to find an admissible approach point within a narrow corridor, rather than reacting to obstacles added later (Figure 23f).

Figure 23a shows a sideview during the test—it is evident that both the UAV and the F1 flag were above ground level. Dimensions visible in Figure 23a,b are defined in Table 1 below.

The test stand serves as the local environment but is not equivalent to the landing pad. A potential landing spot must fit within a circle of 55 cm radius—40 cm for the UAV body and 15 cm as a safety margin. This is smaller than the test stand, enabling flexible scenario setup. No special modifications were made to the test stand. The white protective cloth visible in Figure 20 is part of a separate experiment conducted simultaneously.

The flat surface of the test stand allowed for free placement of obstacles, effectively shaping the landing area. Boxes visible in Figure 23 disrupt the floor’s planarity, prompting the algorithm to find alternative landing sites. These boxes appear as holes in the flat surface to the detection algorithm. Therefore, the collision avoidance algorithm is not required to avoid the boxes. To ensure gradual testing, the collision avoidance range was shortened—boxes were handled by the landing spot detection algorithm, while only the F1 flag (Figure 23a,e) triggered the collision avoidance module.

## 8. Results

### 8.1. Simulation—Landing Accuracy Check

**Figure 24:**
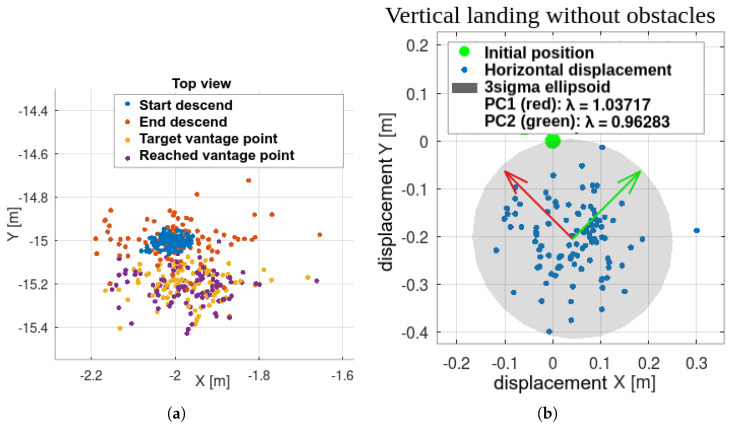
Initial test: vertical landing without obstacles. UAV initiated motion beyond camera range, triggering all autonomous flight stages. Repeatability is evident in (**a**) displacements and (**b**) PCA.

PCA analysis shows no directional preference, but the entire set is translated, indicating a non-zero systematic error. This may be due to modeling inaccuracies or image processing errors. Dislocations are smaller than the UAV dimensions and fall within the safety margin. Thus, the basic landing functionality is validated, and further testing is justified (Figure 24b).

The UAV’s starting position is taken as the origin, and all dislocations are referenced to it.

### 8.2. Simulation—Static Obstacles

**Figure 25:**
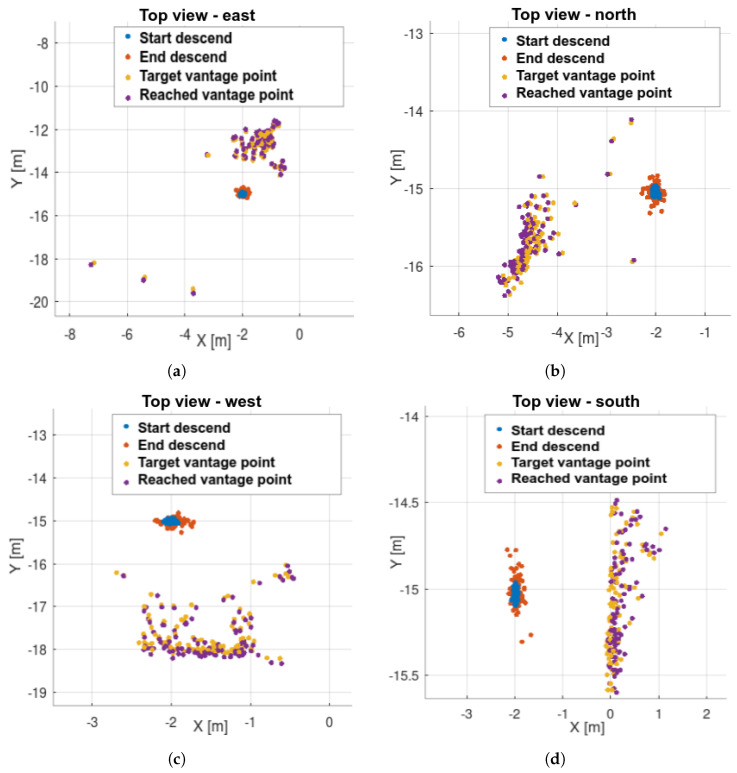
Landing in statically cluttered environment – steps: (**a**) scenario “east”, (**b**) “north”, (**c**) “west”, (**d**) “south”.

**Figure 26:**
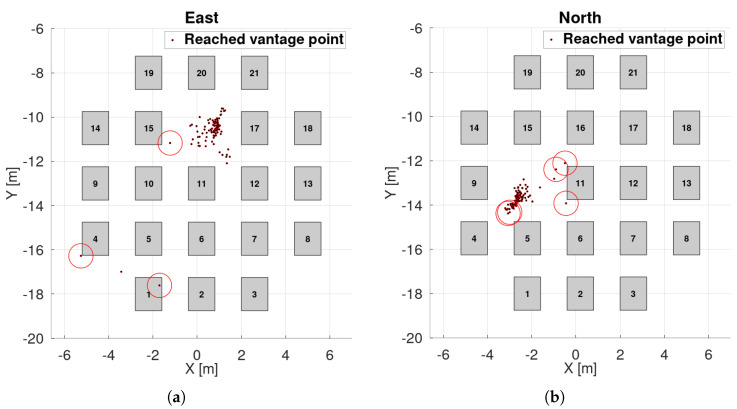

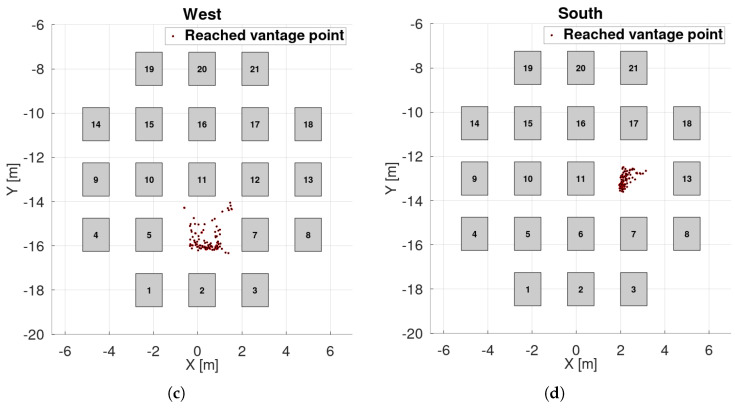
Landing in statically cluttered environment—box placements, reached vantage points and collisions. UAV footprint is treated as the circle with 55 cm radius: (**a**) scenario “east”, (**b**) “north”, (**c**) “west”, (**d**) “south”.

**Figure 27:**
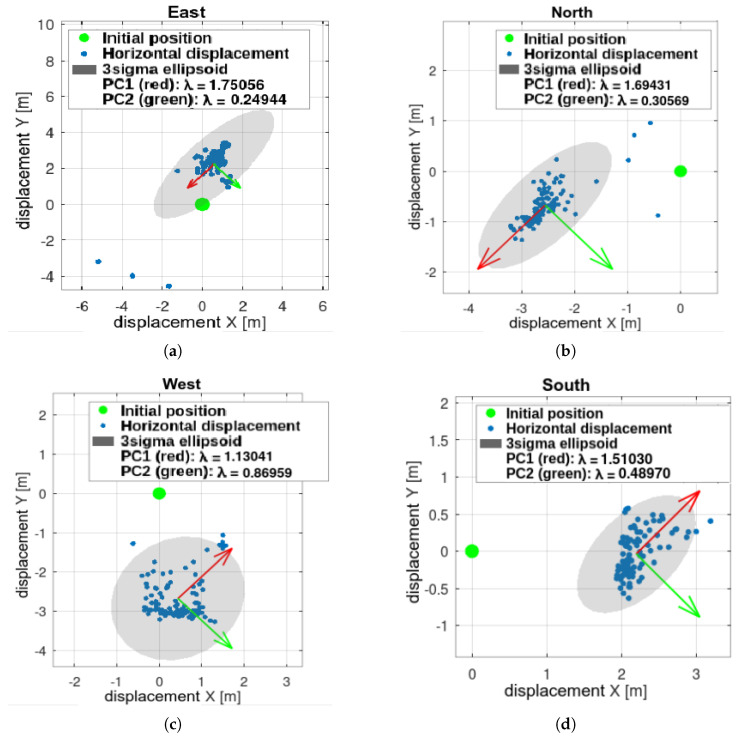
Landing in statically cluttered environment–PCA analysis: (**a**) scenario “east”, (**b**) “north”, (**c**) “west”, (**d**) “south”.

Results are shown in Figure 25, Figure 26 and Figure 27. Landing spots were successfully detected and avoided static obstacles. In this experiment, gimbal motion was active. Landing spots exhibited greater spread and directional preferences, as shown by PCA analysis. One reason for this is the interaction between the environment’s shape and the landing spot selection rule, which favors points closer to the UAV. Combined with other inaccuracies, this leads to points being detected along obstacle edges.

Outliers occurred but were not dominant. Even the outlier points were safe for landing—they were simply detected outside the obstacle zone.

The UAV is modeled as a circle with a 55 cm radius (40 cm for the body and 15 cm for motion inaccuracies). The experiment was designed so that landing directly below the UAV was impossible, as the motion started directly above an obstacle. This implies a 100% failure rate for basic commercial landing methods relying solely on acceleration readings without external sensing.

Success rate is defined as the ratio of landing attempts that maintain a minimum safe distance from obstacles. Results are presented in Table 2 below.

The worst-case scenario yields a 95.14% success rate, compared to 0% for methods without external sensing.

### 8.3. Simulation—Intrusions

In intrusion tests, only the system’s reaction was evaluated—no statistical analysis was performed. Landing began outside the camera range at 12 m AGL. The ground became visible at around 8 m AGL, triggering the second flight stage. With no intrusion detected, the UAV continued descending. An obstacle was detected at around 4 m AGL, prompting the UAV to stop, find a new landing site, and proceed.

**Figure 28:**
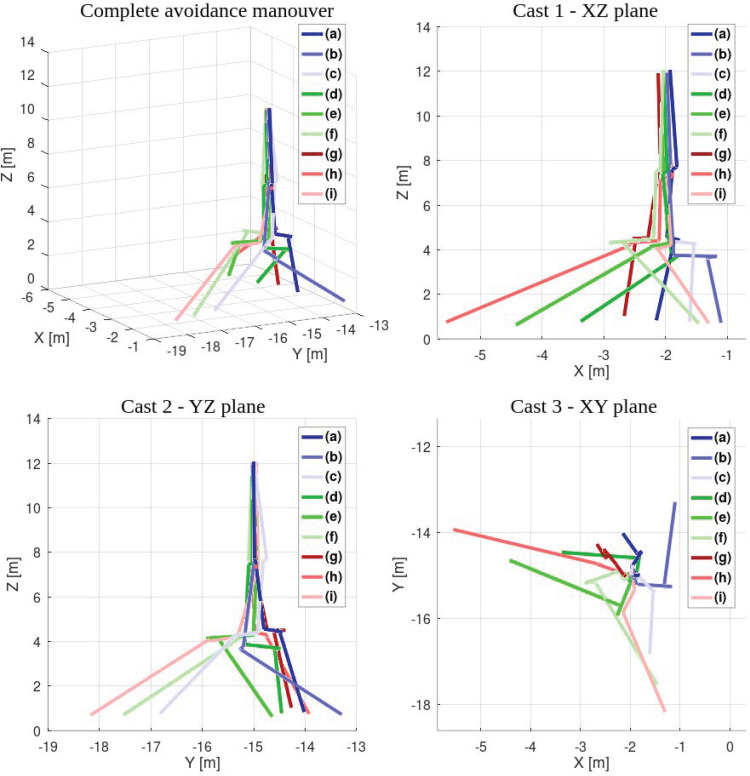
Reactions to the intrusions.

No flight path remained undisturbed, indicating that intrusions from all directions were detected (Figure 28). After obstacle detection, flight paths visibly diverged. Compared to the previous experiment’s cluttered environment, this spread suggests correct reactions—the UAV attempted to move away from the intrusion direction.

### 8.4. Laboratory Results

**Figure 29:**
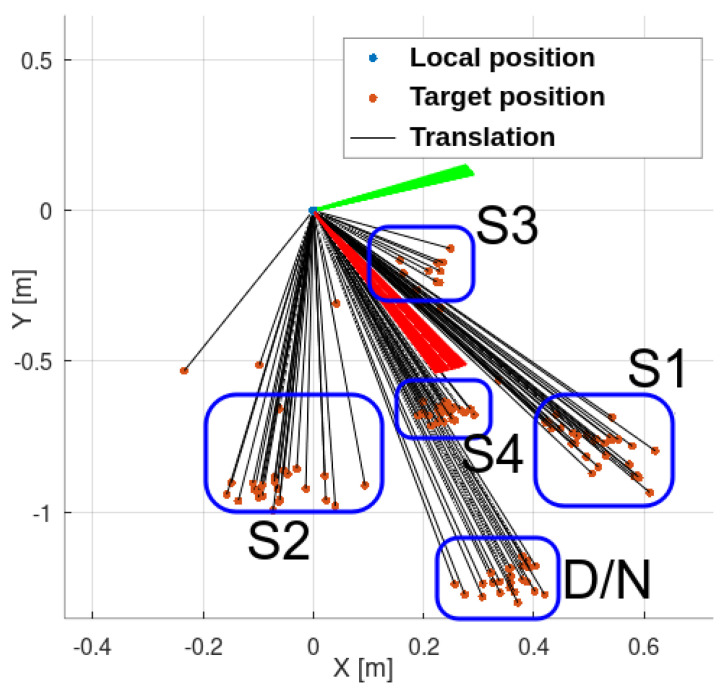
Results of the laboratory test. Repeatability remains evident, consistent with the simulation results. System reactions are clearly observable and identifiable. D/N—scenario day and night, S1–4—scenarios 1–4.

Laboratory tests yielded similar outcomes. The UAV responded to all obstacles, and the collision avoidance module functioned correctly. The tendency to cluster landing points remained consistent with simulation results. A summary is shown in Figure 29.

**Figure 30:**
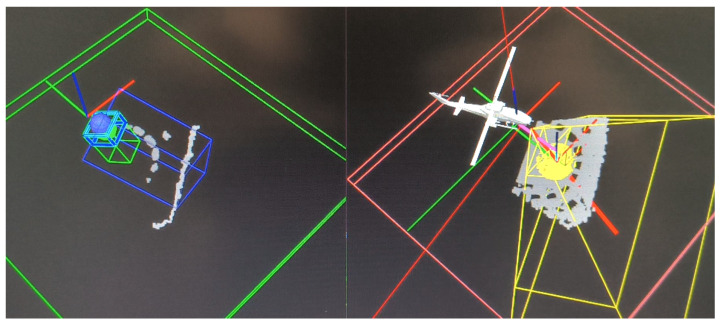
System in operation. The point cloud is divided into two parts: the landing plane (**right**) and non-coplanar points processed by the anti-collision module (**left**).

Figure 30 illustrates how the input point cloud is divided between plane detection (right panel) and collision avoidance (left panel). The small green box on the left indicates the collision avoidance corridor, which was reduced to fit the limited dimensions of the KUKA KUBE test bed. The blue box (optional) shows the UAV’s velocity vector direction.

## 9. Discussion

The experiments demonstrate that a short-range RGB-D camera can safely guide a UAV during both day and night operations. The algorithm defines the UAV’s behavior when flying above the camera’s effective range, and this logic was validated in simulation. Night-time operation without artificial lighting was successfully demonstrated in the laboratory using the real UAV.

Simulations covered all system modules. During these runs, flight altitude was provided by a rangefinder. Thanks to active gimbal control, the camera’s narrow field of view was no longer a limitation—the gimbal continuously tracks the point currently under evaluation, allowing landing sites to be selected anywhere around the UAV. Repeatability and intrusion-response tests confirm that the method works across all azimuths, with candidate points analyzed regardless of their bearing. The range of behaviors considered here exceeds those in previous studies, which often lacked collision avoidance and the ability to operate beyond the depth camera’s range.

Future work will include in-flight outdoor trials, refinement of the landing-site detector for improved robustness under real-world conditions, and tighter time synchronization to ensure each computation uses measurements from the correct instant. The anti-collision module will be redesigned for denser environments, and a more powerful onboard computer is being considered—processing already loads the Jetson Nano to over 80%, even with voxelization.

Currently, all obstacles are treated as static—no segmentation or motion modeling is used. Nevertheless, the system can still respond to dynamically occurring obstacles. Collision avoidance checks for any points present in the flight corridor, and scanning is always active during flight. As shown in the simulation intrusion tests, the UAV can abort the landing procedure when an obstacle appears. Future work on collision avoidance will focus on flight path planning in denser environments and improved obstacle shape analysis.

Dynamic obstacles, such as approaching objects, may be considered in future work. If any point comes too close to the UAV, the system could initiate a retreat to maintain a safe distance. Full scene segmentation is not currently planned, as the emphasis remains on keeping the algorithm lightweight enough for onboard processing.

Real-world conditions such as rain or fog may affect the proposed system if they interfere with the RGB-D camera. Rain could reduce the effectiveness of the RealSense D435i IR projector and introduce noise into the data. Fog is less problematic due to the system’s short-range design, though it could still affect the laser altimeter. Smoke, however, could obscure the ground and render landing spot detection impossible, as neither RGB nor NIR wavelengths can penetrate thick smoke.

As long as data noise from weather or surface conditions is not dominant, filtering strategies can mitigate negative effects. An improved surface model could help distinguish between actual obstacles and noise. Single outliers that do not persist across frames likely indicate noise, while stable outliers or consistent features suggest obstacles.

More advanced filtering and plane segmentation strategies are planned to enhance the system’s robustness in real-world conditions.

Future work will also include a more deliberate gimbal motion strategy. Currently, gimbal movement is driven by tracking the currently evaluated landing-spot candidate and by avoidance behavior. Planned improvements will introduce predefined scanning angles to assess the entire surrounding area before making decisions. This may include stitching planar segments from multiple views to identify suitable landing spots.

Modifications to the landing spot detection strategy are also planned. At present, the algorithm searches for areas with sufficient dimensions. In the future, it will aim to find the largest possible area that is still close to the UAV and meets the dimensional criteria. This change would allow the UAV to maximize its distance from obstacles by utilizing available space more effectively.

The proposed solution differs significantly from most approaches reported in the literature. Therefore, the authors believe that further development of this method is well justified.

## Figures and Tables

**Table 1:** Geometry of the KUKA KUBE test stand and reference frames.

Symbol	Description	Value
*a*	Size of the KUKA KUBE platform plate	250 cm
*b*	Distance from UAV centre to the side with the rail and KUKA robot	110 cm
*c*	Distance from UAV centre to the rear edge of the platform	68 cm
*H*	Height of UAV centre above the plate level	138 cm
*Z*	Height of F1 flag centre above the plate level	84 cm
$\left(X_{\mathrm{UAV}},\;Y_{\mathrm{UAV}}\right)$	Vehicle body coordinate frame axis-aligned with the testing bed	
$\left(X_{\mathrm{PX}4},\;Y_{\mathrm{PX}4}\right)$	Local frame (PX4 position–estimator output)	

**Table 2:** Success rate of landing in cluttered environment.

Scenario	Attempts	Fails	Success Rate
East	103	3	97.10%
North	103	5	95.14%
West	108	0	100%
South	100	0	100%

## Data Availability

The data used to support the findings of this study are available from the corresponding author upon request.

## Abbreviations

The following abbreviations are used in this manuscript:

UAV	Unmanned Aerial Vehicle
ENU	East North Up
NED	North East Down
DTM	Digital Terrain Model
DSM	Digital Surface Model
SBC	Single Board Computer
GPS	Global Positioning System
GNSS	Global Navigation Satellite System
